# Developmental roles and molecular mechanisms of Asterix/GTSF1

**DOI:** 10.1002/wrna.1716

**Published:** 2022-02-02

**Authors:** Jonathan J. Ipsaro, Leemor Joshua‐Tor

**Affiliations:** ^1^ Howard Hughes Medical Institute W.M. Keck Structural Biology Laboratory, Cold Spring Harbor Laboratory Cold Spring Harbor New York USA

**Keywords:** Asterix, germline, GTSF1, piRNA, transposon

## Abstract

Maintenance of germline genomic integrity is critical for the survival of animal species. Consequently, many cellular and molecular processes have evolved to ensure genetic stability during the production of gametes. Here, we describe the discovery, characterization, and emerging molecular mechanisms of the protein Asterix/Gametocyte‐specific factor 1 (GTSF1), an essential gametogenesis factor that is conserved from insects to humans. Beyond its broad importance for healthy germline development, Asterix/GTSF1 has more specific functions in the Piwi‐interacting RNA (piRNA)–RNA interference pathway. There, it contributes to the repression of otherwise deleterious transposons, helping to ensure faithful transmission of genetic information to the next generation.

This article is categorized under:

Regulatory RNAs/RNAi/Riboswitches > RNAi: Mechanisms of Action
Regulatory RNAs/RNAi/Riboswitches > Regulatory RNAs
RNA Interactions with Proteins and Other Molecules > RNA‐Protein Complexes
RNA Interactions with Proteins and Other Molecules > Protein‐RNA Interactions: Functional Implications

## Graphical abstract

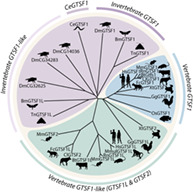

## 1 INTRODUCTION

During metazoan gametogenesis, extensive epigenetic reprogramming occurs in the germline (Iovino, 2014; Surani, 2001; Surani et al., 2007). These changes allow for the transmission of both genetic and epigenetic information, and ultimately allow for the zygotic totipotency required for development in the offspring (Reik & Surani, 2015). Such a dramatic transcriptional reset presents problems, however. Genes that are deleterious and which had been silenced through heterochromatic compaction or DNA methylation can reactivate, threatening the health of the germ cells and consequently the progeny (Bao & Yan, 2012).

A prime example of these damaging genes are transposons, mobile genetic elements that can insert into distant genetic loci and potentially disrupt coding or regulatory regions. Widespread expression of transposons causes genomic instability and therefore must be repressed (Hedges & Deininger, 2007; Saint‐Leandre et al., 2020). In order to protect the germline genome, animals have evolved an adaptive, RNA interference‐based defense system—the Piwi‐interacting RNA (piRNA) pathway—to silence transposable elements (Aravin et al., 2006; Aravin et al., 2007; Girard et al., 2006; Lau et al., 2006). Mutations of genes in this pathway lead to transposon derepression and ultimately cause defects in fertility (Czech et al., 2013; Handler et al., 2013; Li et al., 2021; Muerdter et al., 2013).

After providing an overview of piRNA biology, we detail how the piRNA protein Asterix/Gametocyte‐specific factor 1 (GTSF1) was identified, its roles in development, and the findings that implicate it in piRNA‐directed silencing. This is presented first in the context of mammals followed by that of insects and other model systems. We then describe our current knowledge of Asterix/GTSF1 structural biology and biochemistry and finally pose unanswered questions regarding its action.

## 2 OVERVIEW OF THE piRNA PATHWAY

As mentioned above, the piRNA pathway is a germline‐specific silencing mechanism that is largely dedicated to transposon repression. Like other RNA interference pathways, the central silencing complex is an Argonaute family protein bound to a single‐stranded nucleic acid guide (Liu et al., 2004; Song et al., 2004). These guides ultimately allow for specific and programmable localization of the loaded Argonaute—termed RISC, RNA‐induced silencing complex—using base complementarity of the guide to a nucleic acid target (Hammond et al., 2001; Hutvagner & Zamore, 2002; Martinez et al., 2002; Mourelatos et al., 2002; Verdel et al., 2004).

In the case of the piRNA pathway, Piwi clade Argonautes bind to single‐stranded RNA guides that are 23–32 nucleotides in length (Houwing et al., 2007; Siomi et al., 2011; Vagin et al., 2006). For descriptive purposes, the pathway is customarily divided into two, inter‐related processes: (i) biogenesis and loading of piRNAs and (ii) effector step silencing.

**FIGURE 1:**
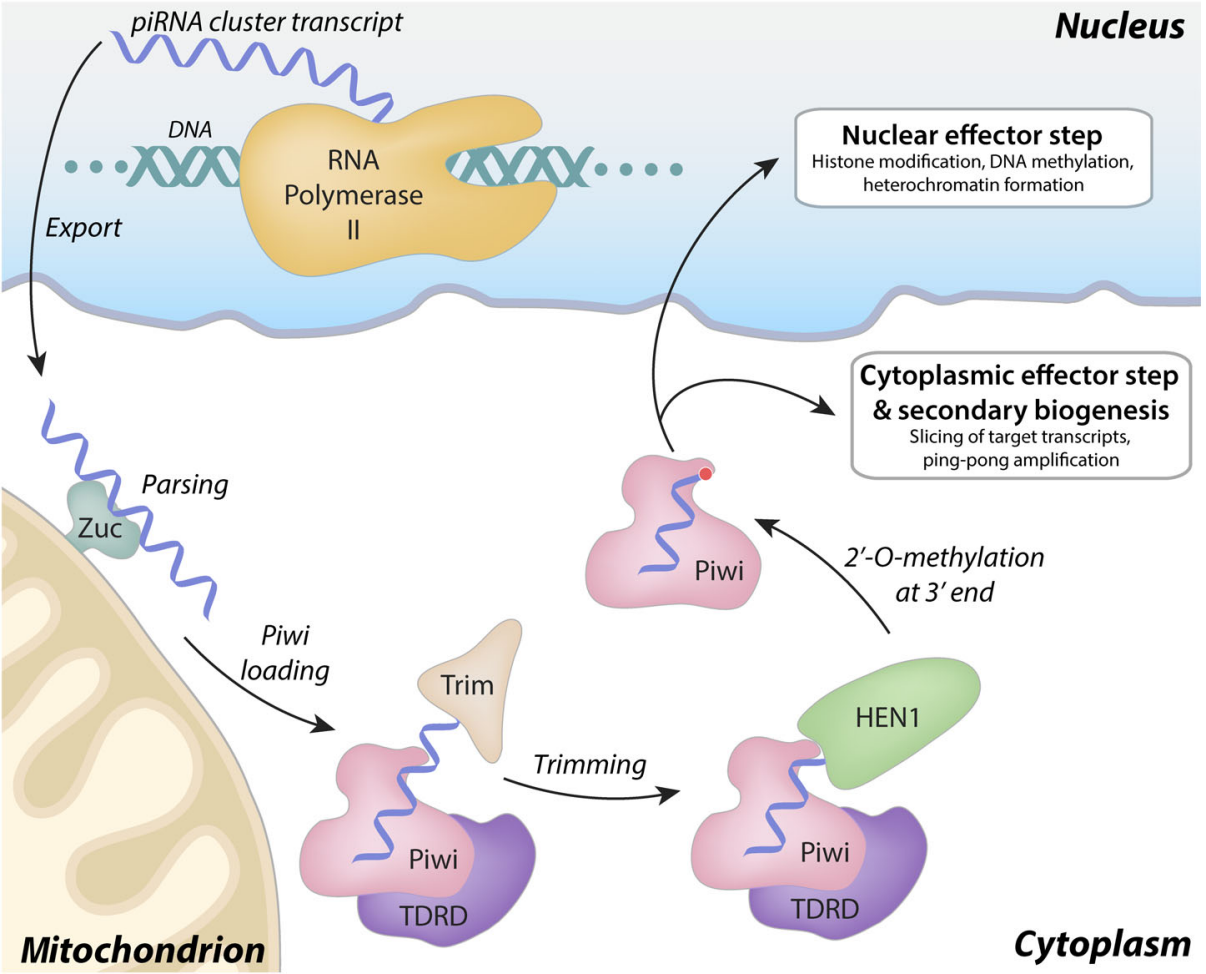
Overview of primary piRNA biogenesis. Primary piRNA biogenesis begins from the nuclear transcription of piRNA clusters, which serve as molecular repositories of silencing targets. These long, multi‐kilobase transcripts are exported from the nucleus, parsed into smaller fragments by Zucchini (Zuc; dark green) and other factors, then loaded into a Piwi clade Argonaute protein (e.g., Piwi in *Drosophila* or MIWI in mouse; pink). The 3′ end of the pre‐piRNA is trimmed by Trimmer/PNLDC1 (Trim; beige) then 2′‐O‐methylated at the 3′ end by HEN1 (light green; methylation indicated by red circle) generating a mature piRISC. This complex can enforce nuclear silencing, cytoplasmic silencing, and/or secondary piRNA biogenesis

Primary piRNA biogenesis (Figure 1) begins with transcription from piRNA clusters: discrete genetic loci that effectively provide a molecular repository of transposons to be silenced (Aravin et al., 2007; Brennecke et al., 2007; Gan et al., 2011). These piRNA cluster transcripts, which are many kilobases in length, are exported to the cytoplasm, processed by endonucleases, then loaded into Argonaute family proteins of the Piwi clade [reviewed in Czech et al. (2018)]. Following loading, the pre‐piRNA is then trimmed by an exonuclease and 2′‐O‐methylated at its 3′ end, resulting in a mature piRISC.

**FIGURE 2:**
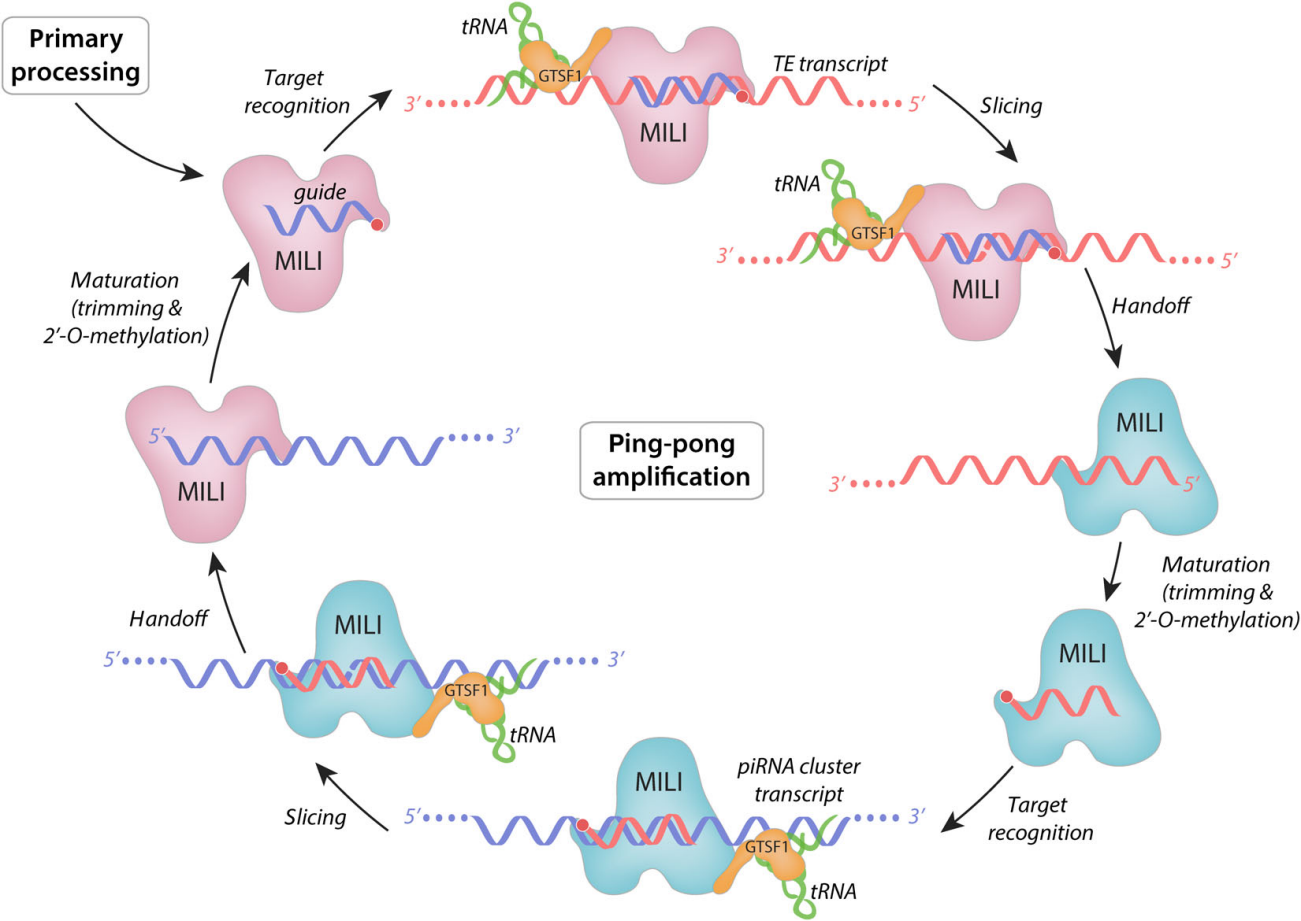
Ping‐pong amplification and secondary piRNA biogenesis in mammals. Mature piRISCs engage with target transcripts (top) and enforce silencing by endonucleolytic “slicing”. This activity is native to mammalian Piwi proteins like MILI, but is potentiated by GTSF1 (Arif et al., 2021). Recent data suggest that recognition of target loci by GTSF1 is accomplished through tRNA‐meditated interactions with GTSF1 and the target transcript (Ipsaro et al., 2021). Subsequently, the sliced target can be loaded into another Piwi clade Argonaute molecule (right), such as another copy of MILI, matured, then used for recognition of complementary transcripts (bottom). Another round of slicing and maturation completes this cycle, generating a pool of piRISCs that adaptatively recognize and silence abundant targets. It should be noted that additional Piwi proteins—including MIWI and MIWI2 in mice—also form piRISCs for silencing. However, those complexes do not seem to participate in ping‐pong to the same extent. Moreover, interactions between sets of Piwi and GTSF1 paralogs do appear to potentiate cleavage universally, although this is the case for GTSF1 with MIWI and MILI (Arif et al., 2021)

Depending on the species and the identity of the Piwi protein, the mature piRISCs can localize to distinct cellular compartments to exert their effects. In *Drosophila*, there are three Piwi proteins: Piwi, Aubergine, and Ago3. While Piwi functions primarily in the nucleus to enforce transcriptional gene silencing, Aubergine (Aub) and Ago3 localize to cytoplasmic foci (termed “nuage” in *Drosophila*), and participate in an adaptive, “ping–pong” amplification cycle to silence transposons post‐transcriptionally (Aravin et al., 2008; Brennecke et al., 2007; Gunawardane et al., 2007; Rozhkov et al., 2013). In mammals, there are three Piwi paralogs (MIWI, MILI, and MIWI2 in mice, for instance), which associate with two, distinct, developmental‐stage specific piRNA pools (Fu & Wang, 2014; Manakov et al., 2015). Each of the mouse homologs can be found in cytoplasmic foci where ping‐pong amplification occurs (Figure 2), with MIWI2 also displaying nuclear localization (Aravin et al., 2008).

**FIGURE 3:**
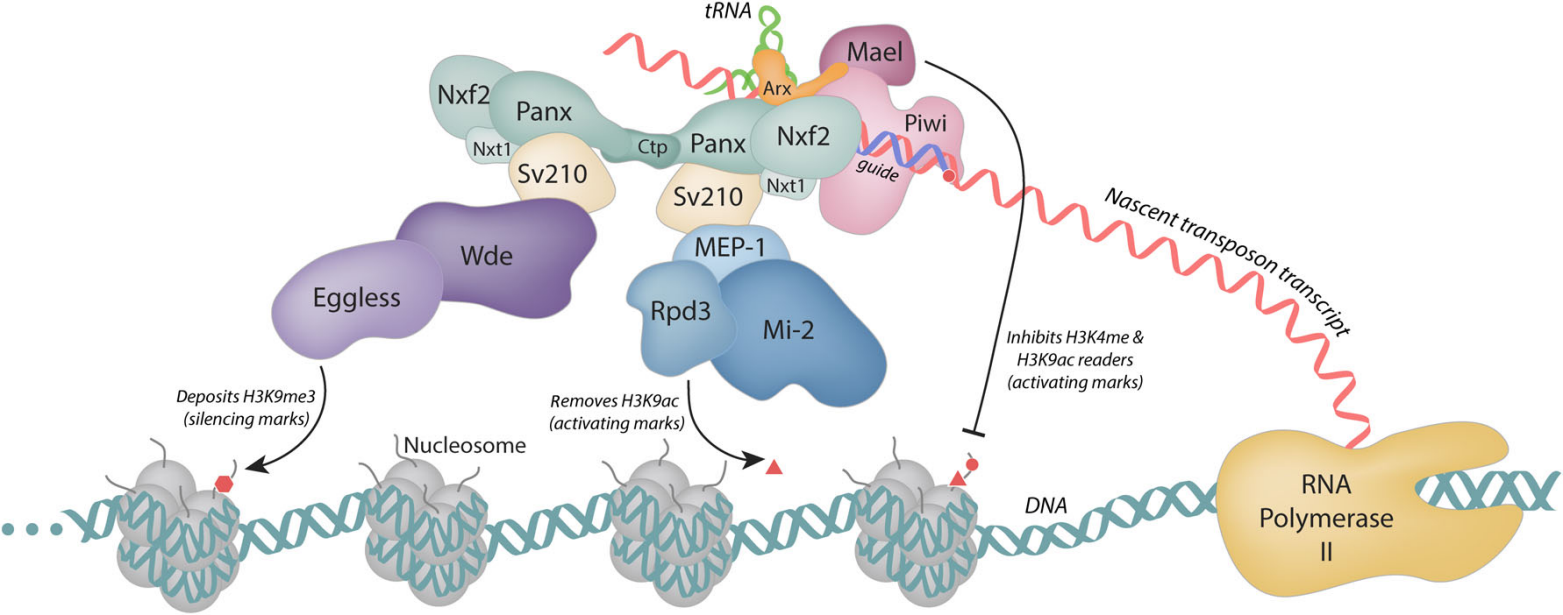
The nuclear piRNA effector step in *Drosophila* leads to transcriptional silencing of transposons. Once a mature piRISC (pink) is localized to the nucleus and engages with its target, other factors are recruited to enforce transcriptional silencing. Asterix/DmGTSF1 (orange) is believed to be a scaffolding molecule in building these assemblies and interacts directly with Piwi. Additional molecules such as Panoramix (Panx), Nxf2, Nxt1, and Ctp (green) interact with Su(var)2–10 (Sv210; beige), which coordinates binding to chromatin modifiers. Eggless, the *Drosophila* homolog of SETDB1, and Wde (purple) deposit histone 3 lysine 9 trimethyl silencing marks. In parallel, the Mi‐2 complex—including MEP‐1 and Rpd3 (blue)—removes histone 3 lysine 9 activating marks. Additionally, Maelstrom (Mael) is recruited to these loci, which inhibits histone 3 lysine 4 methyl and histone 3 lysine 9 acetyl readers. Together, these interactions act to silence transposons co‐transcriptionally and lead to heterochromatin formation at target loci

**FIGURE 4:**
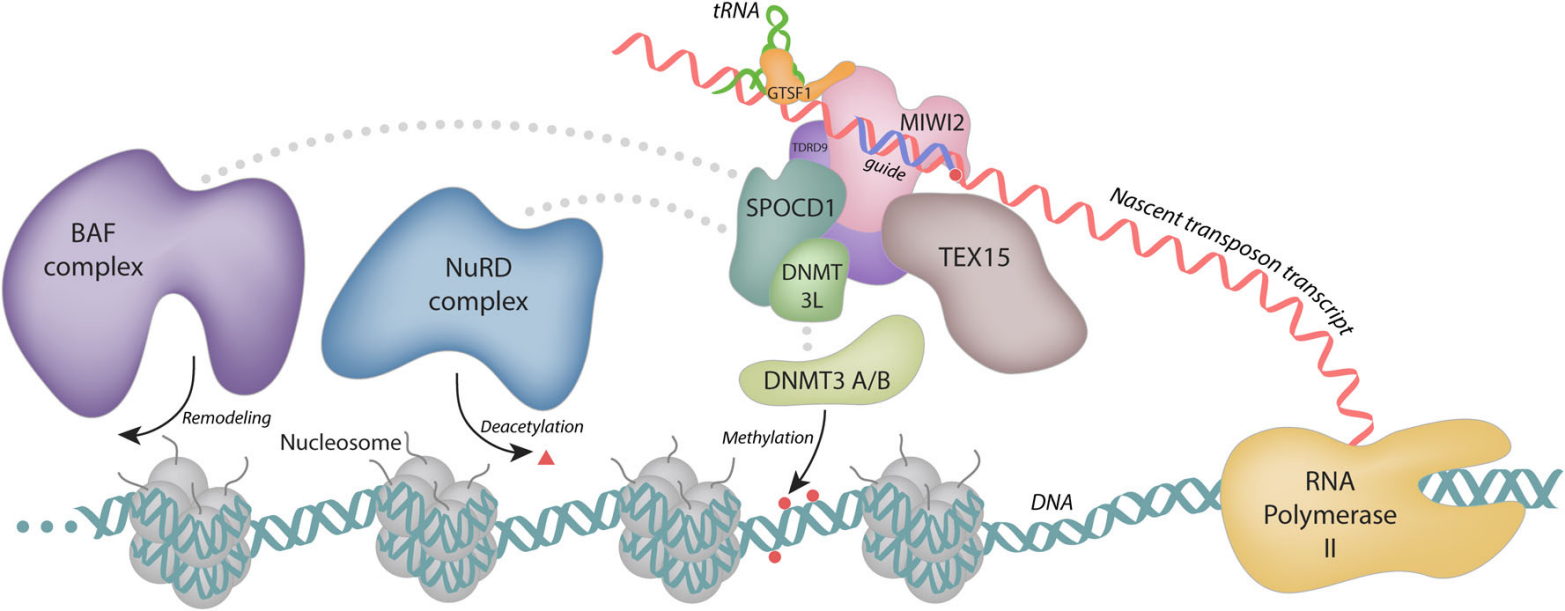
The nuclear piRNA effector step in mammals leads to transcriptional silencing of transposons. The current understanding of nuclear piRNA‐directed silencing in mice is shown as an example for silencing in vertebrates. As is the case in invertebrates, mature piRISC complexes (in this case MIWI2, pink, bound to a piRNA guide) localize to the nucleus, engage with target transcripts, and recruit additional machinery to promote heterochromatin formation. This is likely done in concert with GTSF1, which associates directly with mammalian Piwi proteins such as MIWI2. Factors such as SPOCD1 coordinate interactions with nucleosome remodelers such as the BAF and NuRD complexes (purple and blue, respectively). Methylation of DNA is accomplished by DNMT proteins (green). Together, chromatin remodeling in addition to DNA methylation leads to co‐transcriptional silencing of transposon loci

Regardless of the species and context, piRISCs both associate with and rely upon numerous other factors to effectively enforce silencing (Czech et al., 2013; Handler et al., 2013; Li et al., 2021; Muerdter et al., 2013; Figures 3 and 4). Many of these cofactors, including Asterix/GTSF1, are conserved from flies to humans, though the specifics regarding the particular sequence of events and interactions involved vary. This review focuses on the findings that implicate Asterix/GTSF1 in piRNA‐mediated silencing of retrotransposons and demonstrate its importance in germline development.

## 3 DISCOVERY OF *CUE110/GTSF1* IN MAMMALIAN REPRODUCTION

**FIGURE 5:**
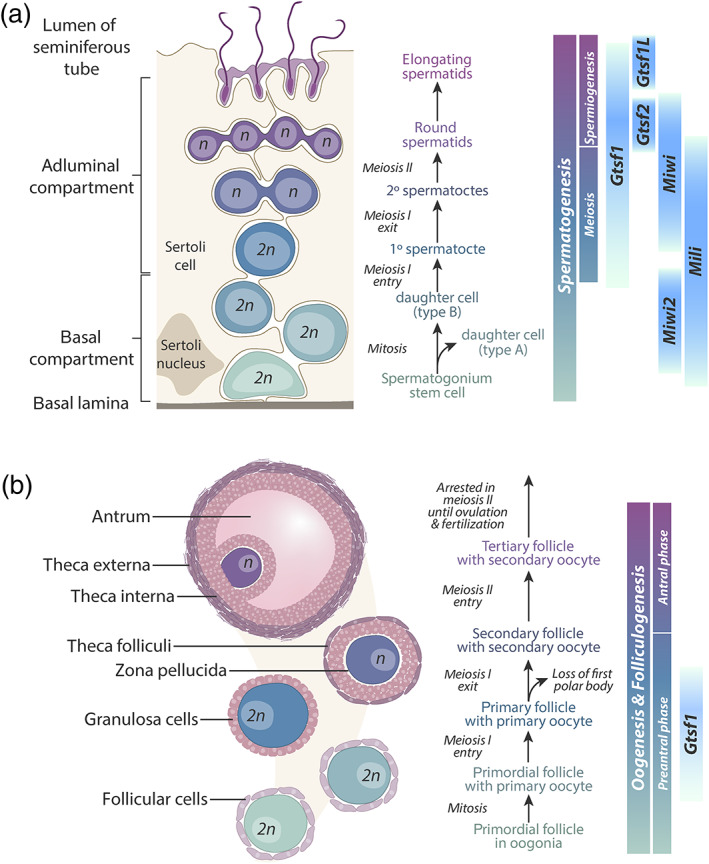
Expression of *Gtsf1* during mammalian germ cell maturation is regulated spatiotemporally. (a) Schematic of germ cell development in male mice. Differentiation of germ cell progenitors begins near the basal lamina and proceeds toward the seminiferous tube lumen (left) while coinciding with gametogenesis (center). Expression of *Gtsf1*, its paralogs, and mouse Piwi proteins (*Miwi*, *Miwi2*, and *Mili*) are indicated (right). *Gtsf1* RNA levels begin preleptotene (early meiosis I), then increase during the round spermatid stage, reach a maximum in late round spermatids, then taper with spermatid elongation. GTSF1 protein is detectable beginning in the pachytene stage of meiosis I. (b) Schematic of germ cell development in female mice. Oocyte maturation involves coordinated gametogenesis along with folliculogenesis (left, center). In situ hybridization indicates that *Gtsf1* expression is restricted to primary oocytes in primordial, primary, and secondary follicles (right)

In mammals, gametes are generated continuously in the male testes and prenatally in female ovaries. These processes—spermatogenesis and oogenesis, respectively—are tightly regulated and coordinate not only mitotic and meiotic cell divisions, but also differentiation and epigenetic reprogramming (Figure 5). Thus, in addition to their medical relevance regarding fertility, germ cells also present a system where the intricacies of gene regulation at transcriptional, post‐transcriptional, translational, and post‐translational levels can be interrogated. Moreover, given the complex orchestration of germline gene expression, cellular differentiation, and tissue development, thorough characterization of novel genes' expression patterns can inform potential molecular functions.

One strategy to identify cell‐specific regulatory factors employs in silico analysis of publicly‐available gene expression datasets, typically from Expressed Sequence Tags (ESTs) or RNA sequencing data. Yoshimura et al. (2007) performed such an analysis in mice and identified 168 ESTs as gametogenesis candidates, termed “Cues” (Computationally obtained Undifferentiated and/or Embryonic stem cell‐specific genes). Further analysis of *Cue110*, revealed germline‐specific expression, being found at moderate levels in unfertilized eggs and ovaries and abundantly in adult mouse testes (Yoshimura et al., 2007). This tissue‐specific expression eventually warranted renaming of the gene to *Gtsf1* (Yoshimura et al., 2007).

## 4 DEVELOPMENTAL TIMING AND CELL‐SPECIFICITY OF *GTSF1* EXPRESSION

In male mice, *Gtsf1* transcription is observed beginning around embryonic day 12.5 (E12.5), remains relatively constant until birth, then plateaus around postnatal day 14 (P14) (Krotz et al., 2009; Yoshimura et al., 2007; Figure 5). Protein levels generally follow this trend, though Gtsf1 protein abundance is markedly higher at 4 weeks of age (and beyond) compared to P14 levels (Yoshimura et al., 2007).

While *Gtsf1* expression in male mice persists through adulthood, it has a limited temporal expression during germ cell differentiation (Figure 5a). In situ hybridization in adult testis sections showed *Gtsf1* expression beginning in preleptotene (early meiosis I) spermatocytes. RNA expression levels increase during the round spermatid stage, reach a maximum in late round spermatids, then decrease with the progression of elongation (Yoshimura et al., 2007). GTSF1 is detectable by immunohistochemistry slightly later, in pachytene (prophase of meiosis I) spermatocytes and round spermatids (Yoshimura et al., 2007). More sensitive follow‐up studies using immunofluorescence have additionally detected GTSF1 protein in prospermatogonia, slightly earlier in development (Yoshimura et al., 2018). GTSF1 expression has not been observed late in sperm maturation and as such is not found in epididymal spermatozoa (Yoshimura et al., 2007). Together, these observations pinpoint *Gtsf1* expression in male mice beginning around E13.5 and being expressed primarily from the pachytene spermatocytes in meiosis I to the round spermatid stage.

Unlike male germline development, mammalian oogenesis occurs prenatally and is arrested in meiosis II until ovulation in adulthood (Figure 5b). In female mice, *Gtsf1* transcription is first detected at low levels around E13.5, increases (Krotz et al., 2009; Yoshimura et al., 2007), then peaks within 2 days of birth (Yoshimura et al., 2007). There are conflicting reports surrounding the protein and RNA expression levels postnatally, however. The initial characterization of GTSF1 expression levels in mice showed that protein levels decrease substantially within 2 weeks after birth, with neither transcripts nor protein being detected in 2‐week‐old mouse ovaries (Yoshimura et al., 2007). The same study also employed in situ hybridization of ovary sections and found *Gtsf1* expression to be restricted to particular stages of differentiation: in primary oocytes in primordial, primary, and secondary follicles (Yoshimura et al., 2007). However, an independently produced report published shortly thereafter described a different temporal expression profile altogether: transcription was detected around E13.5, increased until birth, but then persisted at high levels throughout adulthood in all stages of follicle maturation (Krotz et al., 2009).

Paralleling the findings of the earlier mouse study, transcript levels in human fetal testis and ovaries increased from 8 to approximately 20 weeks of gestation (Huntriss et al., 2017). In ovaries, the highest RNA expression was found in germinal vesicle‐stage oocytes, with a subsequent decrease in meiosis II oocytes (Huntriss et al., 2017). *Gtsf1* was also found during late preimplantation embryos, likely being reactivated after fertilization. This is supported by a blastocyst‐specific transcript variant of *Gtsf1*, which lacks one exon, being detected solely at this developmental timepoint (Huntriss et al., 2017).

Taken together, while there is a potential for some variation, these data suggest that GTSF1 is involved in both male and female gametogenesis in mammals as well as having a role post‐fertilization. In the context of germ cells, *Gtsf1* expression in both males and females is centered on meiosis, a time when maintaining genomic stability is particularly critical.

## 5 ORGANISMAL AND CELLULAR PHENOTYPES UPON LOW EXPRESSION

While *Gtsf1* expression can be detected in both the male and female mammalian germlines, the protein is required for fertility only in males. In fact, while no obvious phenotype was observed in female *Gtsf1*−/− mice, males were sterile despite being otherwise healthy and copulating normally (Yoshimura et al., 2009). Correspondingly, biopsied samples from human males with cryptorchidism (undescended testes) show much weaker GTSF1 expression in patients with other hallmarks of low fertility (Hadziselimovic et al., 2015). The observation that GTSF1 expression persists in females among several species—including mouse (Krotz et al., 2009; Yoshimura et al., 2007), human (Huntriss et al., 2017), cattle, and sheep (Liperis et al., 2013)—implies that it may have beneficial function in oocytes and/or the early embryo. This notion is further supported by a decrease in *Gtsf1* levels in human postovulatory‐aged oocytes (Huntriss et al., 2017; Trapphoff et al., 2016).

In addition to sterility, male mice display obvious gonadal tissue abnormalities (Yoshimura et al., 2009). Histological examination of the testis and epididymis in adult *Gtsf1*−/− mice shows that while Sertoli support cells and spermatogonia are normal, some gonadal cells display enlarged nuclei, scattered chromatin, and undergo apoptosis (Yoshimura et al., 2009). This leads to a dramatic depletion of meiotic cells, with postmeiotic cells—including spermatids and spermatozoa—being absent altogether. These abnormalities were evident around P14, consistent with the involvement of *Gtsf1* in meiosis during spermiogenesis (Yoshimura et al., 2009).

Collectively, these findings demonstrate that GTSF1 is required for mammalian male fertility. Its absence causes widespread gonadal abnormalities including pre‐pachytene meiotic defects, a lack of postmeiotic cells, and apoptotic cell death. There is evidence for potential GTSF1 involvement in oogenesis and early development, but its precise role has yet to be determined.

## 6 INVOLVEMENT IN TRANSPOSON SILENCING

The apoptosis seen in *Gtsf1* −/− male mice, as well as the analysis of several meiotic progression markers by immunofluorescence, suggested an accumulation of DNA damage (Yoshimura et al., 2009, 2018). These observations, along with the expanded nuclei, scattered chromatin, and sterility are reminiscent of *Miwi2* (Carmell et al., 2007) and *Mael* (Soper et al., 2008) mutant mice—in addition to piRNA mutant phenotypes in flies (see below)—and suggested GTSF1 could similarly be involved in the piRNA pathway.

With this in mind, transposon levels were compared between *Gtsf1* −/− and +/− testes. It was found that expression levels of two representative transposable elements, Line‐1 (a LINE, non‐LTR retrotransposon) and IAP (an LTR retrotransposon) were significantly higher in the mutant background in mouse prospermatogonia (Yoshimura et al., 2009). Correspondingly, Line‐1 retrotransposons were also found to be derepressed in the human male patients with cryptorchidism mentioned above (Hadziselimovic et al., 2015). Together, these findings directly link *Gtsf1* with retrotransposon silencing in mammals. It follows that the cause of infertility upon GTSF1 depletion, therefore, is due to widespread genomic instability caused by transposon derepression.

## 7 SUBCELLULAR LOCALIZATION AND BINDING PARTNERS

As mentioned, *Gtsf1* is mainly expressed in pachytene (meiosis I) spermatocytes and round spermatids (meiosis II) (Yoshimura et al., 2007). There, it localizes to cytoplasmic granules (termed “pi‐bodies” and “piP‐bodies” in mammals, depending on their composition (Aravin et al., 2009)) and this is likely the main site of GTSF1 action (Yoshimura et al., 2009). It should be noted, however, that since its original characterization, GTSF1 has also been found in E17.5 prospermatogonia where it localized not only to cytoplasmic granules but also to nuclei (Yoshimura et al., 2018).

Given its role in transposon silencing, it was speculated that GTSF1 could be involved in piRNA‐directed silencing. In fact, the granular cytoplasmic localization of GTSF1 observed early in its characterization suggested that it may co‐localize with piRNA components such as MAEL, MILI, MVH, and/or TDRD1 (Aravin et al., 2009; Yoshimura et al., 2009). Findings in *Drosophila* around this time supported this notion (Donertas et al., 2013; Muerdter et al., 2013; Ohtani et al., 2013) and prompted further inspection of the protein's localization and binding partners.

Immunofluorescence data from wild‐type male mice indicated that GTSF1 completely co‐localized with the piP‐body components MIWI2 and TDRD9 (Yoshimura et al., 2018). The specific importance of GTSF1 in piP‐bodies was further bolstered by observations in *Gtsf1* homozygous null mice where piP‐body components MIWI2, MAEL, and TDRD9 are mislocalized, while pi‐body components (such as MILI, MVH, and TDRD1) localize normally (Yoshimura et al., 2018).

Pull‐down experiments using recombinant, tagged GTSF1 with testis lysate, showed that GTSF1 physically associates with piRNA pathway components MIWI, MIWI2, MILI, TDRD1, and TDRD9, and that the central region of GTSF1 (residues 90–123) was sufficient for inclusion in these complexes (Yoshimura et al., 2018; see Section 16, below). This was further validated using GTSF1 IP‐MS which again found each of the components. Correspondingly, direct binding has been shown for GTSF1 with TDRD9 by reconstitution using recombinantly expressed proteins. Interestingly, treatment with RNase A limited MIWI2 binding, but did not affect MIWI, MILI, or TDRD, suggesting that RNA interactions may be required in some contexts for GTSF1 action (Yoshimura et al., 2018).

## 8 ROLES IN MAMMALIAN piRNA BIOLOGY

In addition to *Gtsf1*−/− phenocopying piRNA pathway mutants, binding piRNA pathway proteins, and being critical for retrotransposon silencing, additional molecular findings in mice point to GTSF1 as a bona fide piRNA pathway member. Moreover, the implication of GTSF1 in piRNA biology was further informed by work done in *Drosophila*, as will be discussed below.

Lack of GTSF1 in mouse prospermatogonia leads not only to retrotransposon derepression but also to a decrease in sense and antisense LINE‐1 piRNAs (Yoshimura et al., 2018). While MILI‐bound piRNAs remain intact in *Gtsf1*‐null mice when assessed in immunoprecipitates, MIWI2‐bound piRNA levels are strikingly ablated (Yoshimura et al., 2018). Correspondingly, size distributions of small RNA libraries prepared in *Gtsf1*−/− versus +/− mice skew roughly 2 nucleotides smaller (the preferred substrate MILI is ~26 nucleotides whereas that for MIWI2 is ~28 nucleotides), and the fraction of secondary piRNAs—distinguished by an enrichment of adenosine at the tenth nucleotide—is lower. Intriguingly, while GTSF1 properly localizes to piP‐bodies in MIWI2 (a piP body component generally involved in secondary piRNA processing) knockout mice, it fails to do so in a MILI (a pi‐body component that is involved in primary piRNA processing) null context (Yoshimura et al., 2018). Together, these findings suggest that GTSF1 does not affect primary piRNA biogenesis yet is involved in the generation of the secondary piRNAs required for robust transposon silencing (Yoshimura et al., 2018). This is further bolstered by observations that *Gtsf1*−/− mice fail to direct piRNA‐mediated cleavage of specific ping‐pong targets (Yoshimura et al., 2018).

The piRNA pathway limits transposable element expression not only through post‐transcriptional silencing but also via genomic control. piRNA‐dependent methylation of transposon promoter regions provides proactive transposon repression upstream of transcription. Indeed, reduced methylation levels of retrotransposon regulatory regions have been reported in testes in MIWI2‐, MILI‐, and MAEL‐null mice compared to their heterozygotic littermates. Similar observations have been made in *Gtsf1*−/− mice for both LINE and LTR retrotransposons, further implicating GTSF1 as a piRNA pathway component (Yoshimura et al., 2009).

## 9 MAMMALIAN PARALOGS OF GTSF1

Two related genes, *Gtsf1L* and *Gtsf2*, are also found in mammals and show similar expression patterns to *Gtsf1* in male mice (Figure 5a). *Gtsf1L's* first wave of expression is slightly earlier in mouse development (E7.5–E15.5) compared to *Gtsf1*. *Gtsf2*, on the other hand, is slightly later (E15.5–E18.5). Both of these orthologs are expressed again postnatally, beginning around P20 and P14, respectively (Takemoto et al., 2016).

Interestingly, during spermatogenesis, GTSF1L and GTSF2 showed specific and sequential expression, with GTSF1L being expressed highest in early elongating spermatids and GTSF2 being expressed highest in late round spermatids. Expression outside of these stages was markedly weaker—so much so that upon their initial characterization, the authors suggest these proteins as cell stage markers (Takemoto et al., 2016).

GST pull‐down experiments using recombinantly produced GTSF1L or GTSF2 with mouse testis lysate showed that these proteins can bind MIWI, MILI, and TDRD1. These interactions are mediated by one of the N‐terminal Zn‐finger domains rather than through the C‐terminal tail, as seen for GTSF1. Interestingly, these interactions are RNA‐dependent for GTSF1L, but seem dispensable for GTSF2 binding (Takemoto et al., 2016).

Despite these associations, there were no obvious organismal or molecular phenotypes upon knockout of *Gtsf1L* and/or *Gtsf2*. Male mice showed normal development, typical germline differentiation, and transposon silencing even in the double‐null context (Takemoto et al., 2016).

## 10 DISCOVERY OF ASTERIX

Unlike the discovery of GTSF1 in mammals which sought to identify gametogenesis‐related proteins, Asterix/DmGTSF1 (the ortholog in *Drosophila*) was found through the identification of genes involved in retrotransposon silencing. In 2013, transgenic RNAi screens by multiple groups identified a previously uncharacterized protein, *CG3893*, as a critical component for transposon silencing in the female germline in *Drosophila melanogaster* (Czech et al., 2013; Handler et al., 2013; Muerdter et al., 2013; Ohtani et al., 2013). Moreover, a P‐element insertion into the CG3893 coding sequence provided independent genetic evidence for the protein's involvement in transposable element silencing, with soma‐ and germline‐specific transposons being derepressed and female flies displaying only rudimentary ovaries and sterility (Donertas et al., 2013).

Owing to the protein's <20 kDa size but potent phenotype when knocked out, the protein was named *Asterix* after the small‐but‐mighty French comic book hero (Muerdter et al., 2013). Since its identification in *Drosophila*, other insect orthologs have also been studied, most notably in the silkworm *Bombyx mori* (*BmGtsf1*) owing to this organism's expanding role as a piRNA biology model system. Regardless of the species, similar to its role in mammals, Asterix/GTSF1 is involved in germ cell development in insects with its expression restricted to the gonads.

## 11 PHENOTYPE

Unlike in mammals where *Gtsf1* seems to be primarily involved in male gametogenesis, in *Drosophila* data focus on female fertility owing to the fact that Asterix expression is highest in the ovary (Larkin et al., 2021). Upon knockout of *Asterix*, female flies are completely sterile, lay no eggs, and display small, malformed ovaries. Outside of these germline deficiencies, female flies appear normal (Donertas et al., 2013; Muerdter et al., 2013; Ohtani et al., 2013).

In silkworm, *BmGtsf1* leads to gametogenesis defects in both males and females, with testes and ovarioles being highly atrophied (Chen et al., 2020). Interestingly, CRISPR/Cas9‐induced mutation of *BmGtsf1* induced partial sex reversal in genotypically female silkworm, indicating that this protein can have roles not only in gametogenesis, but also sex determination (Chen et al., 2020).

## 12 LOCALIZATION AND BINDING PARTNERS

In contrast to its cytoplasmic localization in mammals, in *Drosophila*, Asterix is enriched in the female germline and somatic cell nuclei. Immunofluorescence data from multiple groups indicated that within the nucleus Asterix/DmGTSF1 co‐localizes with a sub‐pool of Piwi (Donertas et al., 2013; Muerdter et al., 2013; Ohtani et al., 2013), reminiscent of GTSF1 interaction networks in mouse. This was followed by the observation that Piwi knockdown reduces Asterix/DmGTSF1 nuclear enrichment and suggested their involvement in the same complex (Donertas et al., 2013). Even more compellingly, when Piwi is mutated to remove its N‐terminal nuclear localization signal, Asterix/DmGTSF1 relocalizes to the nuage with Piwi, indicating that Piwi localization dictates that of Asterix/DmGTSF1 in vivo (Donertas et al., 2013).

**FIGURE 6:**
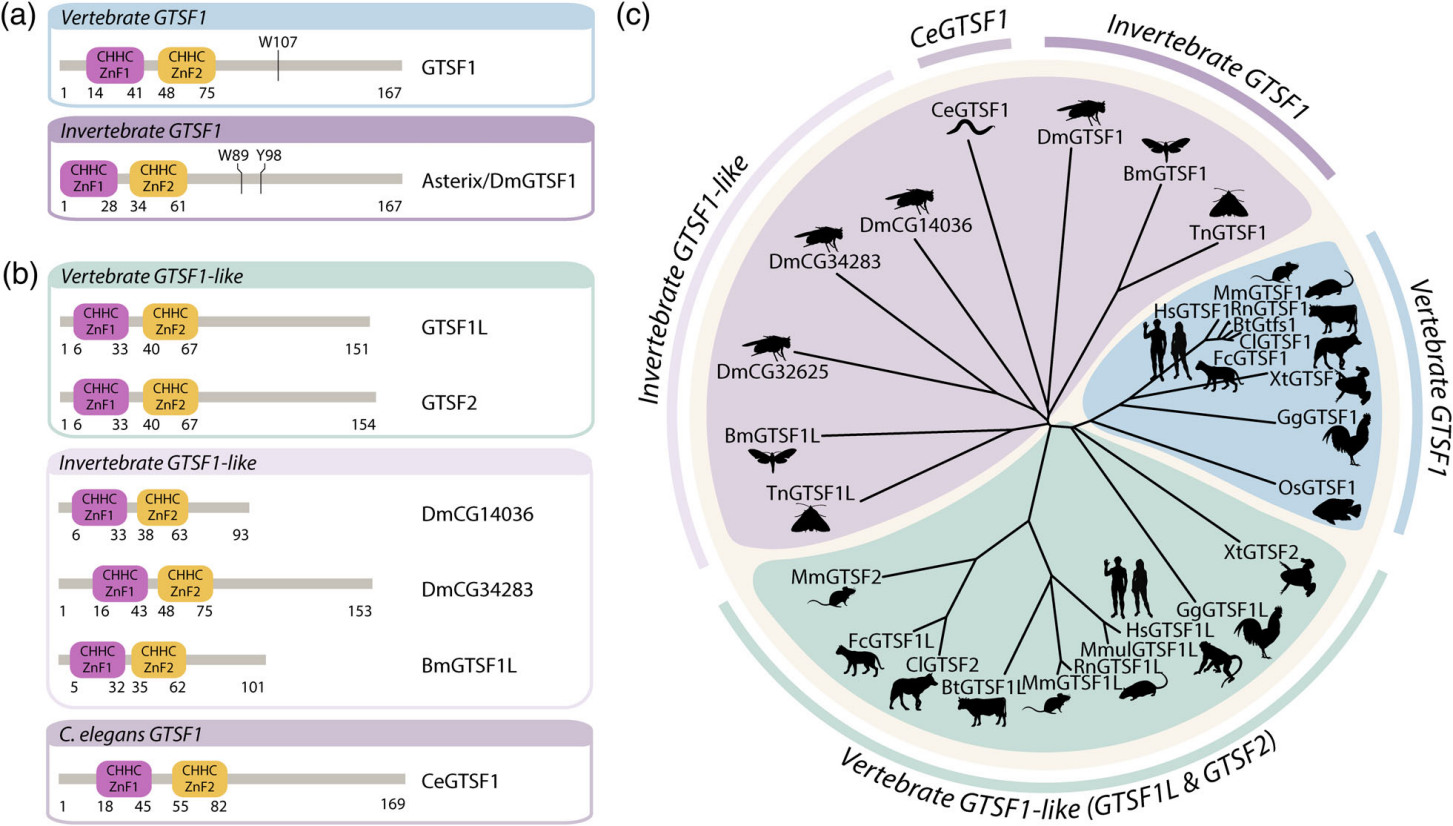
Molecular and evolutionary comparison of Asterix/GTSF1 homologs. All Asterix/GTSF1 homologs are relatively small proteins, less than 20 kDa in size, and generally possess two CHHC zinc finger domains near their N‐terminus. (a) Domain architecture schematics of invertebrate and vertebrate GTSF1 homologs. In addition to their N‐terminal zinc finger domains, both vertebrate GTSF1 (top; light blue) and invertebrate (bottom; light purple) homologs have been shown to interact with their cognate Piwi proteins via aromatic residues (indicated). (b) Domain architecture schematics of other homologs. Similar domain architectures are found for GTSF1‐like proteins in vertebrates (lower left; light green), *C*. *elegans* GTSF1 (top left; purple), and other invertebrates (right; dark purple. (c) Phylogenetic analysis. Multiple sequence alignment suggests close relationships among vertebrate GTSF1 orthologs (light blue), with greater variability among GTSF1‐like orthologs (light green). Even greater differences are noted between invertebrate homologs. Species abbreviations: Bm, *Bombyx mori* (domestic silk moth); Bt, *Bos taurus* (cattle); Ce, *Caenorhabditis elegans* (nematode worm); Cl, *Canis lupus familiaris* (domestic dog); Dm, *Drosophila melanogaster* (fruit fly); Fc, *Felis catus* (domestic cat); Gg, *Gallus gallus* (chicken); Hs, *Homo sapiens* (human); Mmul, *Macaca mulatta* (rhesus macaque); Mm, *Mus musculus* (house mouse); On, *Oreochromis niloticus* (Nile tilapia); Rn, *Rattus norvegicus* (brown rat); Tn, *Trichoplusia ni* (cabbage looper); Xt, *Xenopus tropicalis* (western clawed frog)

Analysis of Asterix‐containing complexes by IP‐MS from cell extracts and in vitro pull‐down assays using recombinantly expressed, tagged Asterix/DmGTSF1 confirmed its association with Piwi (Donertas et al., 2013; Ohtani et al., 2013). This persisted in the presence of RNase A, indicating that their interaction is either RNA‐independent or that any RNA involved is protected or highly structured (Ohtani et al., 2013). The interaction site of Asterix/DmGTSF1 with Piwi was then mapped using pull‐down assays to the central portion of GTSF1 (residues 83–115; Donertas et al., 2013), with certain aromatic residues being critical (Figure 6a). This is in agreement with the binding region defined for mouse GTSF1 with MIWI2‐and MILI‐containing complexes (Yoshimura et al., 2018).

As mentioned, in *Bombyx*, *BmGtsf1* is important for both male and female gametogenesis. There, it is expressed in the gonads, albeit at higher levels in ovaries than testes (Chen et al., 2020). Similar to observations in *Drosophila*, BmGTSF1 is expressed in both the germline and somatic support cells within the ovary (Chen et al., 2020). In contrast, however, BmGTSF1 localizes to both the nucleus and cytoplasm in spite of the fact that the corresponding Piwi protein, BmSIWI is exclusively nuclear (Chen et al., 2020). Male silkworms show BmGTSF1 expression restricted to the spermatogonium, with similar distributions of both BmSIWI and BmVasa (Chen et al., 2020). BmGTSF1 and BmSIWI reciprocally co‐immunoprecipitate each other, demonstrating they are present in the same complex as in *Drosophila* and mammals (Chen et al., 2020).

## 13 ROLES IN THE RNA REGULATION IN INSECTS

In *Drosophila*, piRNA pathway mutants typically display phenotypes of female sterility, ranging from defects in egg morphology to nearly absent ovaries. Besides phenocopying piRNA mutants and binding to Piwi, Asterix/DmGTSF1 knockdown or knockout produces molecular hallmarks that implicate it as a critical piRNA silencing factor.

Two of the three labs that identified Asterix/DmGTSF1 as a piRNA pathway member did so using genome‐wide knockdown screens that probed transposon derepression using reporter constructs (Czech et al., 2013; Handler et al., 2013; Muerdter et al., 2013), while the third employed a candidate approach that followed from the findings of mouse GTSF1 published earlier (Ohtani et al., 2013). Not only were transposons highly upregulated upon Asterix/DmGTSF1 knockdown, but this effect was also specific to transposable elements rather than globally affecting cellular transcription and/or heterochromatin formation (Donertas et al., 2013). Asterix/DmGTSF1 is also involved in other regulatory mechanisms such as piRNA‐mediated regulation of splicing (Teixeira et al., 2017), further implicating it as a piRNA factor.

When Asterix/DmGTSF1 is depleted, not only are reporter constructs activated, but rather retrotransposons are widely derepressed, particularly for LTR transposons (Donertas et al., 2013; Ipsaro et al., 2021; Muerdter et al., 2013; Ohtani et al., 2013). This occurs without major disruption to the levels of Piwi proteins (Piwi, Aub, and Ago3) or piRNAs themselves (Donertas et al., 2013; Muerdter et al., 2013; Ohtani et al., 2013). Strikingly, transposon expression (Donertas et al., 2013; Muerdter et al., 2013; Ohtani et al., 2013) and Pol II occupancy (Donertas et al., 2013) were highly correlated among Asterix/DmGTSF1‐ and Piwi‐depleted cells, with transposable element derepression occurring even more rapidly for Asterix/DmGTSF1 than Piwi knockdowns (Donertas et al., 2013). Finally, H3K9me3 silencing marks, which are ultimately deposited at transposon loci in functional nuclear piRNA‐directed silencing, are similarly lost in Asterix/DmGTSF1 and Piwi knockouts (Donertas et al., 2013; Muerdter et al., 2013; Ohtani et al., 2013).

Similar observations regarding transposon derepression have also been made in *Bombyx*, but it should be noted that BmGTSF1 is also required in spermatogenesis (Chen et al., 2020). Even more interestingly, BmGTSF1 is important for the production of some piRNAs—most notably *Fem* piRNAs which silence the sex‐determining gene *BmMasc* (Chen et al., 2020). Reminiscent of findings in mice, global piRNA levels are slightly reduced and piRNA size distributions skew slightly in *BmGtsf1* knockouts (Chen et al., 2020). Given that BmGTSF1 has some cytoplasmic localization in silkworms (whereas it is exclusively nuclear in flies), this observation suggests that GTSF1‐related proteins can exert different effects that correspond to the cellular compartment in which they are found.

Collectively, these studies provide a compelling framework, establishing Asterix/DmGTSF1 as a piRNA player that exerts its effects downstream of primary piRNA biogenesis.

## 14 PARALOGS IN INSECTS

Whereas mammals have three GTSF1‐paralogs, *Drosophila* have four (Asterix/DmGTSF1/CG3893, and three uncharacterized proteins: CG14036, CG32625, and CG34283) (Muerdter et al., 2013; Ohtani et al., 2013). Each of these shows expression in OSS cells (a tissue culture model derived from *Drosophila* ovaries) (Muerdter et al., 2013) and germline‐specific moderate to high expression in the ovary (Graveley et al., 2011). Similar to the findings in mice, of these paralogs only Asterix/DmGTSF1 seems critical for transposon silencing as knockdown of the others affected neither piRNA nor transposon levels in OSS (Muerdter et al., 2013; Ohtani et al., 2013).

## 15 PARALOGS IN OTHER SPECIES

To date, the majority of studies directly focused on Asterix/GTSF1 have employed mammalian and insect model systems. One notable exception is work done in the nematode worm *Caenorhabditis elegans* which possesses a single homolog, CeGTSF‐1.

Similar to its mammalian orthologs, CeGTSF‐1 is enriched in germline cytoplasm in perinuclear granules (Almeida et al., 2018). During larval development, *gtsf‐1* RNA levels are highest during the L4 and young adult stages, coinciding with germline development (Almeida et al., 2018). These findings alone would suggest a role for CeGTSF‐1 in gametogenesis like that found in other animals, however, this surprisingly is not the case. Homozygous mutants lacking *gtsf‐1* are fertile and develop normally (Almeida et al., 2018). These mutants exhibit neither transposable element derepression nor changes in piRNA‐like 21U‐RNA profiles (Almeida et al., 2018). Rather, CeGTSF‐1 is important for potentiating the RRF‐3‐containing ERIC complex that is responsible for the biogenesis of 26G interfering RNAs (Almeida et al., 2018). The same study implicated the CHHC zinc finger domains as being responsible for mediating protein–protein (rather than protein–RNA) interactions, reminiscent of findings for mouse GTSF1L and GTSF2 (Almeida et al., 2018). Interestingly, this suggests that some GTSF1 orthologs—while still involved in multivalent RNAi pathway interactions—may function entirely as a protein scaffold. Such a role has been observed in the RNA‐induced transcriptional silencing (RITS) complex protein Stc1 in *S*. *pombe* (Bayne et al., 2010; He et al., 2013) which, although phylogenetically unrelated to GTSF1, has a similar domain architecture to GTSF1 (Figure 6a,b) with N‐terminal zinc finger domains and an unstructured C‐terminus (Almeida et al., 2018).

The role of GTSF1 has also been described in fish and prawns where it is relevant for aquaculture. In Nile tilapia (*Oreochromis niloticus*), *OnGtsf1* is specifically expressed in female oogonia and oocytes, and is a marker for sex determination (Tao et al., 2018). MrGtsf in freshwater prawns was found to be similarly specific to the female germline (Jiang et al., 2019). At present, these findings in aquatic life do not yet establish a definitive molecular role for GTSF1, but are highly suggestive that similar mechanisms to those in insects and mammals are utilized.

In summary, GTSF1 homologs have thus far shown consistent germline‐specific expression. The majority of these proteins seem to be involved in transposable element silencing that occurs in an RNA‐dependent fashion, though some have been adapted for other RNA silencing mechanisms.

## 16 PROTEIN PROPERTIES

Regardless of the species, GTSF1 homologs are small, ~19 kDa proteins. They display a simple domain architecture comprising two, N‐terminal CHHC zinc fingers and a C‐terminus that is likely unstructured (Figure 6a,b; Andreeva & Tidow, 2008). Evolutionary analysis of GTSF1 has detected recognizable GTSF orthologs not only in many metazoans, but also in some protozoans (Figure 6c). This conservation is derived almost exclusively from the zinc finger domains, with the C‐terminus of the protein being highly variable. Notably, no orthologs have been reported in plants or fungi (Andreeva & Tidow, 2008).

Some insights into the molecular basis of GTSF1 interactions can be gleaned from a broader analysis of CHHC zinc fingers in general. These small, roughly 40 amino acid domains are monomeric, independently folded, and identifiable by a CPX_4_HX_9_HX_3_C motif (Andreeva & Tidow, 2008). Zn^2+^ is bound by these domains with 1:1 stoichiometry (Andreeva & Tidow, 2008) and their global structure resembles that of their C2H2 zinc‐finger cousins (Tidow et al., 2009). CHHC zinc finger domains are found in three protein groups present exclusively in eukaryotes: spliceosomal U11‐48K proteins, tRNA methyltransferases, and gametocyte‐specific factors (Andreeva & Tidow, 2008). Interestingly, each of these has demonstrated direct binding to structured RNA substrates (Ipsaro et al., 2021; Tidow et al., 2009; Wilkinson et al., 2007), which suggests a shape‐dependent—rather than sequence‐dependent—mode of nucleic acid binding. Characterization of the spliceosomal U11‐48K CHHC domain described salt‐sensitive protein–RNA interactions indicating that, at least for U11‐48K, ionic interactions were the driver of association (Tidow et al., 2009). More detailed biophysical analysis in the same study suggested that U11‐48K binding was perhaps stabilizing particular RNA–RNA interactions (Tidow et al., 2009).

**FIGURE 7:**
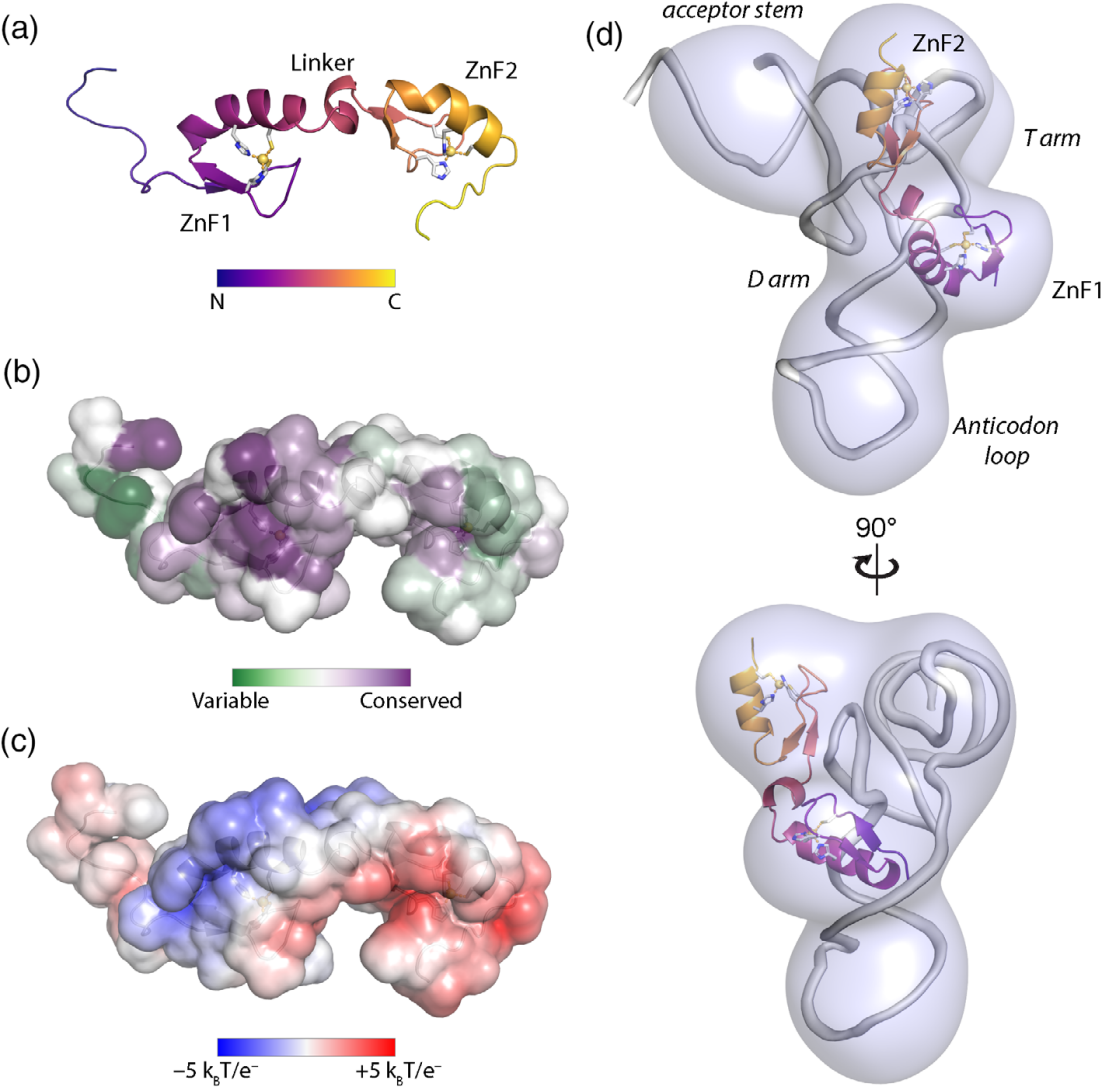
Molecular features of mouse GTSF1. (a) Ribbon diagram of mouse GTSF1 residues 1–80, determined by NMR spectroscopy. Mouse GTSF1 presents two CHHC zinc finger folds that each bind Zn^2+^ with 1:1 stoichiometry. These domains are connected by a short, α‐helical‐containing linker. (b) Conservation analysis of mouse GTSF1. Using the same sequence alignments as in the phylogenetic tree (Figure 6c), conservation scores were mapped onto the mouse GTSF1 surface. The CHHC zinc‐coordinating residues were absolutely conserved in both domains, while the first zinc finger exhibits higher conservation more broadly. (c) Electrostatic surface of mouse GTSF1. A positively charged ridge is situated on ZnF1, largely coincident with the region of high conservation. Basic residues along this ridge are necessary for RNA binding in vitro. (d) Cryo‐EM reconstruction of mouse GTSF1 in complex with tRNA. Mouse GTSF1 interacts directly with tRNA and is positioned such that ZnF1 interacts with the tRNA D arm. ZnF2 extends toward the tRNA acceptor stem

Correspondingly, multiple groups have reported the CHHC zinc fingers of Asterix/DmGTSF1 to be critical for its function. Mutation of zinc‐coordinating residues fails to rescue knockdown of the endogenous protein in OSS cells although nuclear localization and Piwi binding remain unaffected (Muerdter et al., 2013; Ohtani et al., 2013). Moreover, the solution structure of the tandem CHHC zinc finger domains of mouse GTSF1 revealed a highly consistent fold architecture when comparing the GTSF1 zinc finger domains to each other and to the structure of the CHHC domain U11‐48K (Figure 7a; Ipsaro et al., 2021; Tidow et al., 2009). The two CHHC zinc fingers are flanked by unstructured regions and are connected by a flexible, α‐helix containing linker. A relatively conserved (Figure 7b) and positively charged (Figure 7c) surface on the first zinc finger mediates RNA‐binding interactions. This binding site is primarily situated on the α‐helical portion of the first CHHC zinc finger, similar to the location determined for U11‐48K binding dsRNA (Ipsaro et al., 2021; Tidow et al., 2009). Interestingly, while this binding surface is generally conserved among GTSF1 orthologs, the basic residues for mediating RNA‐interactions are not found in *C*. *elegans* GTSF1. This notable exception, combined with the sequence‐based phylogeny (Figure 6c), prompts speculation that gene duplication events led to various, RNA‐binding, GTSF1 paralogs while the RNA‐binding activity was lost in *C*. *elegans*.

Similar to observations with zinc finger mutations, removal of the C‐terminal half of Asterix/DmGTSF1 fails to rescue knockdown in OSS cells (Donertas et al., 2013). Additionally, in both mice and flies, coprecipitation experiments have implicated the central portion of the molecule in Piwi binding (Donertas et al., 2013; Ohtani et al., 2013; Yoshimura et al., 2018). Together, these interaction studies suggest modular functionality for GTSF1, with the first CHHC zinc finger mediating RNA interactions and the C‐terminus binding to Piwi proteins.

## 17 MOLECULAR UNDERPINNINGS OF ASTERIX/GTSF1 INTERACTIONS

More recently, studies on Asterix/GTSF1 have shifted toward revealing its mechanistic contributions to piRNA biology. The genetic and molecular studies described above established that in both mammals and insects Asterix/GTSF1 acts in the piRNA effector step and is not responsible for primary piRNA biogenesis.

To better place Asterix/DmGTSF1 action in the piRNA effector step, tethering experiments that physically direct piRNA players to transposon loci have been employed. These experiments showed that in *Drosophila*, some factors such as Panoramix (Onishi et al., 2020; Sienski et al., 2015; Yu et al., 2015) or Maelstrom (Onishi et al., 2020) can induce silencing of target loci whereas Piwi or Asterix/DmGTSF1 cannot. This indicates that Asterix/DmGTSF1 acts upstream of Panoramix and Maelstrom. Correspondingly, loss of Asterix/DmGTSF1 causes a reduction in Piwi–Maelstrom and Piwi–Panoramix interactions, suggesting that Asterix/DmGTSF1 is needed for assembling other factors on Piwi (Onishi et al., 2020). These findings could indicate that Asterix/DmGTSF1 may lock Piwi in a target‐bound state that is required for effective silencing (Donertas et al., 2013; Ipsaro et al., 2021). Similar assertions have been made in mammalian systems, where it has been posited that mouse GTSF1 is required for MIWI2‐piRISCs to stabilize and/or grasp target RNAs (Yoshimura et al., 2018). Additionally, a recent preprint suggests that mouse GTSF1 is a factor that potentiates mouse Piwi proteins' catalysis, increasing target cleavage by orders of magnitude (Arif et al., 2021).

In addition to its interactions with Piwi proteins, RNA interactions are also critical to Asterix/GTSF1 function (Arif et al., 2021; Ipsaro et al., 2021). Studies of recombinant mouse GTSF1 were the first to show robust and specific co‐precipitation GTSF1 with RNA, which were specifically identified as tRNAs. Similar analysis in both mouse and *Drosophila* cell culture systems reproduced this finding, linking Asterix/GTSF1 to tRNAs directly (Ipsaro et al., 2021).

These observations drew attention to the long‐established dependence of LTR retrotransposon expression on host tRNAs [reviewed in Mak and Kleiman (1997)]. This connection—from Asterix to tRNAs to LTR transposons—is reinforced by genetic observations that LTR retrotransposons are disproportionately affected by Asterix/GTSF1 depletion (Donertas et al., 2013; Ipsaro et al., 2021; Muerdter et al., 2013; Ohtani et al., 2013). Moreover, the same preprint that indicates mouse GTSF1 potentiation of piRISC catalysis also reported an increase in enzymatic activity for sponge GTSF1 with sponge Piwi (EfPIWI) in a tRNA‐containing buffer (Arif et al., 2021). Together, these associations suggest that GTSF1 could be enhancing piRNA‐directed silencing is through tRNA‐directed transposon recognition.

## 18 CURRENT MECHANISTIC MODEL

The CHHC zinc fingers and the C‐terminal tail of Asterix/GTSF1 are involved in distinct interactions (RNA‐binding and Piwi‐binding, respectively), and the necessity for both of these has been demonstrated by numerous studies (Arif et al., 2021; Donertas et al., 2013; Ohtani et al., 2013). Along with the findings that Asterix/GTSF1 specifically binds structured RNAs, such as tRNAs (Figure 7d), and that tRNAs share an evolutionary history with LTR retrotransposons, one model of Asterix/GTSF1 action involves multivalent interactions that add specificity and/or stability to piRISC‐target interactions and enhances otherwise weak piRISC activity (Figures 2, 3, 4).

In *Drosophila*, where Asterix/DmGTSF1 is localized to the nucleus, the data support the notion that Asterix/DmGTSF1 is required for downstream factors like Panoramix or Maelstrom to be recruited to piRISCs (Figure 3). In this context, Asterix/DmGTSF1 could assist in piRISC recognition of target loci, particularly those corresponding to LTR retrotransposons transcripts. Subsequently, Asterix/DmGTSF1 may serve as scaffolding molecule, allowing association of downstream piRNA effectors once “validated” targets have been bound.

In mammals, Piwi proteins such as MIWI, MIWI2, and MILI are expressed with characteristic developmental timing and subcellular localizations. Presently, mouse GTSF1 action in nuclear silencing via MIWI2 (Figure 4) is expected to parallel the mechanism for *Drosophila* Asterix/DmGTSF1, described above. However, when localized to cytoplasmic MIWI‐containing granules, piRISCs act differently. Here, they participate in the piRNA effector step by slicing transposon transcripts and simultaneously generating secondary piRNAs (Figure 2). In this context, MmGTSF1 is believed to increase MIWI‐directed RNA cleavage by more than 100‐fold in a manner that is dependent on both RNA binding and GTSF1–MIWI interactions (Arif et al., 2021). This boost in MIWI activity could result from enhanced piRISC localization to targets through MIWI–GTSF1–RNA interaction networks, independent localization of mouse GTSF1 to target loci followed by triggering piRISC cleavage, or both.

Finally, data from other species such as silkworms, corroborate many of these observations. While there are clearly nuances to Asterix/GTSF1 action across species and cellular compartments, the underlying mechanism seems to employ RNA‐binding in concert with piRISC binding as a means to specify, stabilize, and/or enhance piRNA pathway attributes.

## 19 CONCLUSION

In just over the last decade, Asterix/GTSF1 has been identified and characterized in multiple species. These initial studies have described the fundamental role of this protein in development and reproduction and provided molecular links between Asterix/GTSF1, the piRNA pathway, and retrotransposons.

Many exciting questions follow from these seminal works. First, while piRISCs achieve target specificity from their piRNA guides, the interplay between target context and Asterix/GTSF1 involvement remains to be detailed directly. Second, it appears that GTSF1 interacts with a particular subpool of Piwi proteins. While it may be that GTSF1 recognizes target‐bound piRISCs, this has not been formally demonstrated and it is entirely possible that GTSF1 may in fact initiate target recognition and recruit piRISCs rather than the converse. Additionally, if GTSF1 is using tRNA or tRNA‐like elements to recognize silencing targets, it has not been established how it specifically recognizes those elements only in the context of a transposon. tRNAs are highly abundant and only a fraction of the cellular tRNA pool is involved in retrotransposon reverse transcription. Could Asterix/GTSF1 be specifically recognizing tRNAs that are engaged with LTR transposons, and if so, are auxiliary factors needed for recognition as well? Related to that, does the nature of these interactions change depending on the species and cellular compartment? Finally, it is not understood how Asterix/GTSF1 interacts molecularly with piRISCs to enhance their silencing efficacy. Are conformational changes induced upon piRISC engagement with Asterix/GTSF1? If so, are those changes directly required for recruitment of downstream factors, and if so, do those factors engage with Asterix/GTSF1, Piwi, or both?

The essentiality of Asterix/GTSF1 in fertility along with its connections to piRNA biology, tRNAs, retrotransposon silencing, sex determination, and cell differentiation, bear relevance for many biological topics. Ongoing studies of piRNA‐mediated silencing, along with improvements in sequencing, informatics, and structural methods hold promise for revealing additional, intriguing, molecular underpinnings that underlie Asterix/GTSF1 activity.

## CONFLICT OF INTEREST

The authors declare no conflicts of interest in association with this work.

## AUTHOR CONTRIBUTIONS

**Jonathan Ipsaro:** Conceptualization (lead); visualization (lead); writing – original draft (lead); writing – review and editing (equal). **Leemor Joshua‐Tor:** Funding acquisition (lead); supervision (lead); writing – review and editing (equal).

## RELATED WIREs ARTICLE

Small noncoding RNAs and male infertility

## Data Availability

Data sharing is not applicable to this article as no new data were created or analyzed in this study.
